# Lymphoma combined with *Tropheryma whipplei* infection: A case report and literature review

**DOI:** 10.1097/MD.0000000000044611

**Published:** 2025-09-19

**Authors:** Langqing Xu, Dan Li

**Affiliations:** a Department of Respiratory and Critical Medicine, The First Hospital of Jilin University, Changchun, Jilin Province, China.

**Keywords:** lung infection, lymphoma, Whipple trophoblast

## Abstract

**Rationale:**

Whipple disease is exceptionally rare in patients with lymphoma in China, particularly when presenting with pulmonary symptoms as the initial manifestation. Respiratory involvement is atypical for Whipple disease and may easily be overlooked, especially in patients receiving targeted drug therapy for lymphoma. In such cases, it can be challenging to distinguish between disease progression, drug-induced pneumonia, and infectious etiologies, as the clinical and radiological findings often overlap.

**Patient concerns:**

A 39-year-old patient with lymphoma and persistent fever did not respond well to the broad-spectrum antibiotics.

**Diagnoses:**

In contrast to traditional pathological examination or tissue periodic acid–Schiff staining, the patient was diagnosed using metagenomic next-generation sequencing of pulmonary alveolar lavage fluid.

**Interventions:**

Symptomatic treatment, including anti-infective agents, cough relief, and expectorants, was administered. Bronchoscopy and lung puncture were performed to determine the cause of the disease.

**Outcomes:**

Following the confirmation of *Tropheryma whipplei* by metagenomic next-generation sequencing, targeted antimicrobial therapy was likely initiated. The patient’s symptoms, including the persistent fever, subsequently resolved, indicating a positive response to the appropriate treatment.

**Lessons:**

Whipple trophic pneumonia is an opportunistic infection with low incidence. It typically presents with atypical clinical signs and symptoms, making diagnosis challenging based on the imaging findings. It is often misdiagnosed, particularly in patients receiving immunosuppressants. Gene detection in the lung lavage fluid can aid in diagnosis. This case report discusses a patient with lymphoma and Whipple disease, while also reviewing current literature on the bacterium to enhance clinician awareness of its complexities.

## 1. Introduction

Whipple barrier organism, a rod-shaped aerobic gram-positive facultative pathogenic bacterium, is ubiquitously distributed in the external environment. Initially characterized by George Hoyt Whipple in 1907,^[1]^ this microorganism underwent important taxonomic reclassification following the development of effective antibiotic therapies, the advancement of electron microscopy, and the subsequent application of PCR techniques, culminating in its formal designation as *Tropheryma whipplei* in 2001.^[1]^ Extensive research has established its etiological role in Whipple disease, a chronic multisystemic infectious disorder characterized by recurrent manifestations.^[2]^ The disease predominantly affects the gastrointestinal tract and the articular and central nervous systems, with additional presentations including localized infections such as infective endocarditis.^[3]^ Notably, pulmonary involvement remains a rare clinical manifestation. In this case report, we present a diagnostically challenging case of *T. whipplei* infection, accompanied by a comprehensive analysis of diagnostic and therapeutic approaches.

## 2. Patient information

### 2.1. Clinical findings

A 39-year-old woman was admitted to the First Hospital of Jilin University hospital on October 13,2023 with fever, cough, and a small amount of white sputum for 6 days, with no obvious triggers, and a maximum temperature of 39.4°C, without chills, chest pain, shortness of breath, or hemoptysis. A lung CT scan revealed scattered inflammatory lesions and interstitial changes in all lobes of the lungs, as well as ground-glass nodular shadows in the upper-lobe of the right lung. The patient was admitted to the outpatient clinic with a diagnosis of lung infection. The patient had a history of lymphoma for 2 years and was regularly treated with targeted drugs (tocilizumab). After admission, her vital signs stabilized, and blood gas analysis revealed a partial oxygen pressure of 89 mm Hg. A specialized examination showed that the lungs were clear to percussion bilaterally, her respiratory sounds were thick in both lungs with no dry or wet rales, and no pleural friction sounds detected.

### 2.2. Timeline

#### 2.2.1. *Diagnostic assessment*

**Figure 1.**
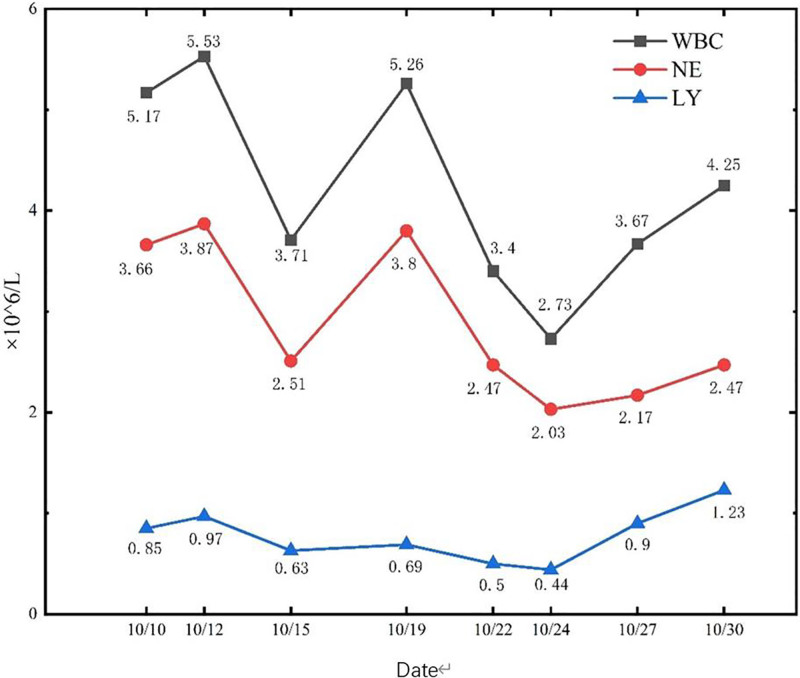
Trend of white blood cells, neutrophils, and lymphocytes.

**Figure 2.**
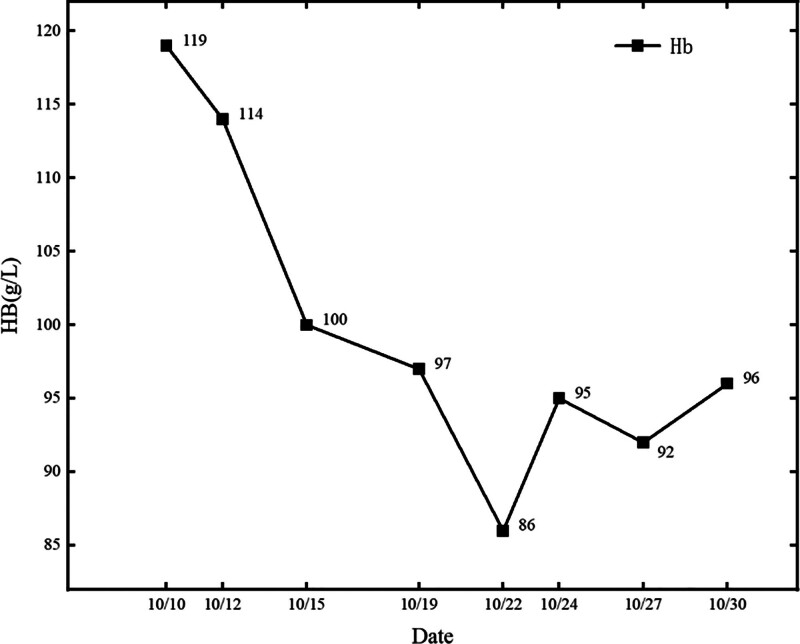
Haemoglobin trend graph.

**Figure 3.**
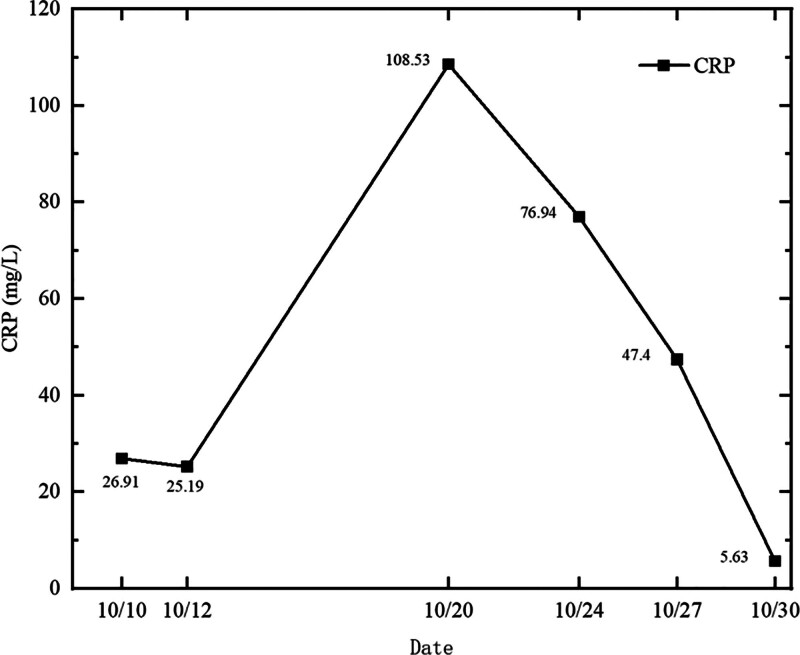
Trend chart of C-reactive protein levels.

**Figure 4.**
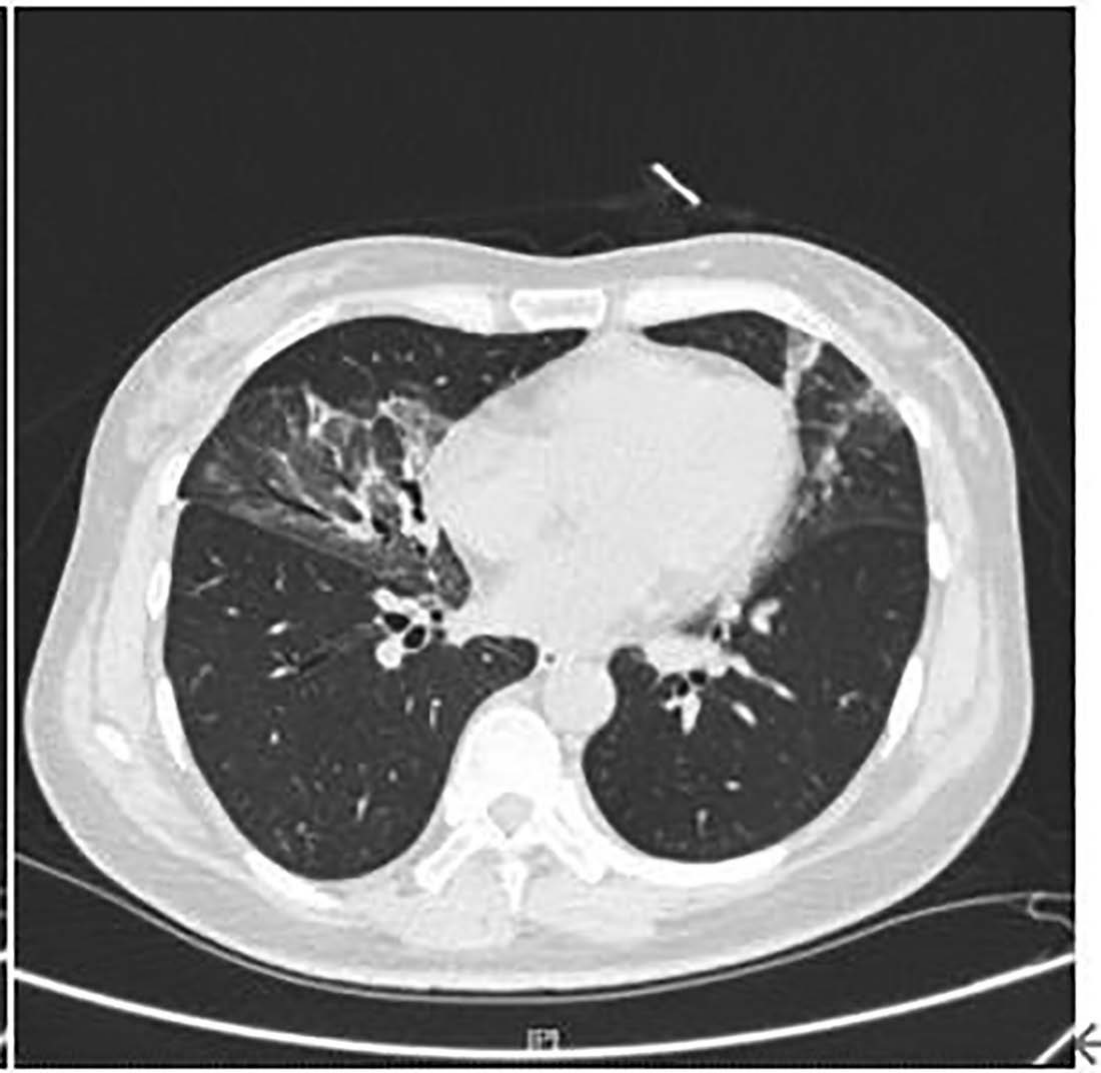
Patient’s pre-admission lung CT on October 12, 2023. CT = computed tomography.

**Figure 5.**
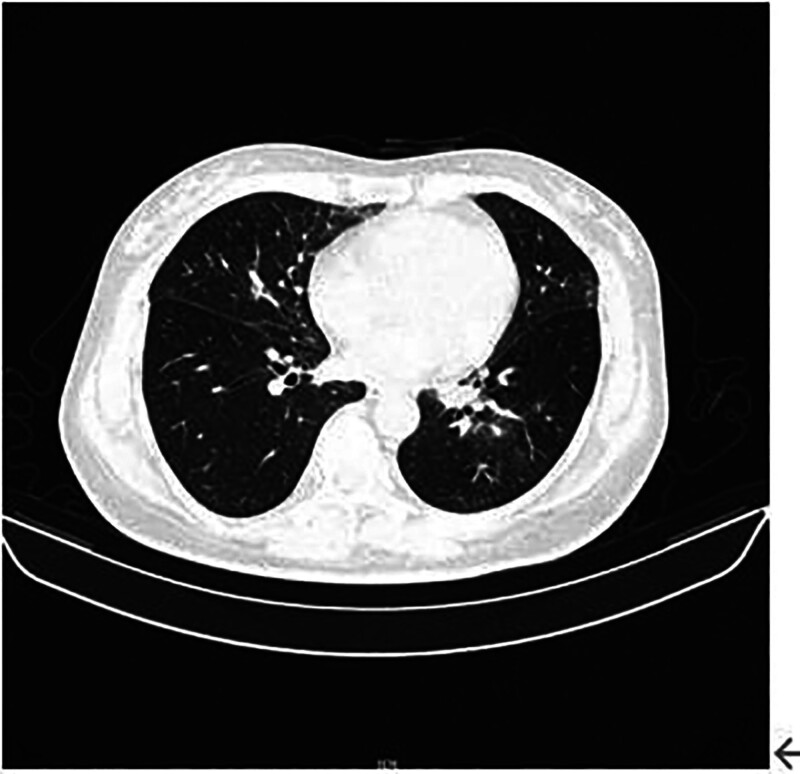
Patient was reexamined using pulmonary CT on October 18, 2023. CT = computed tomography.

**Figure 6.**
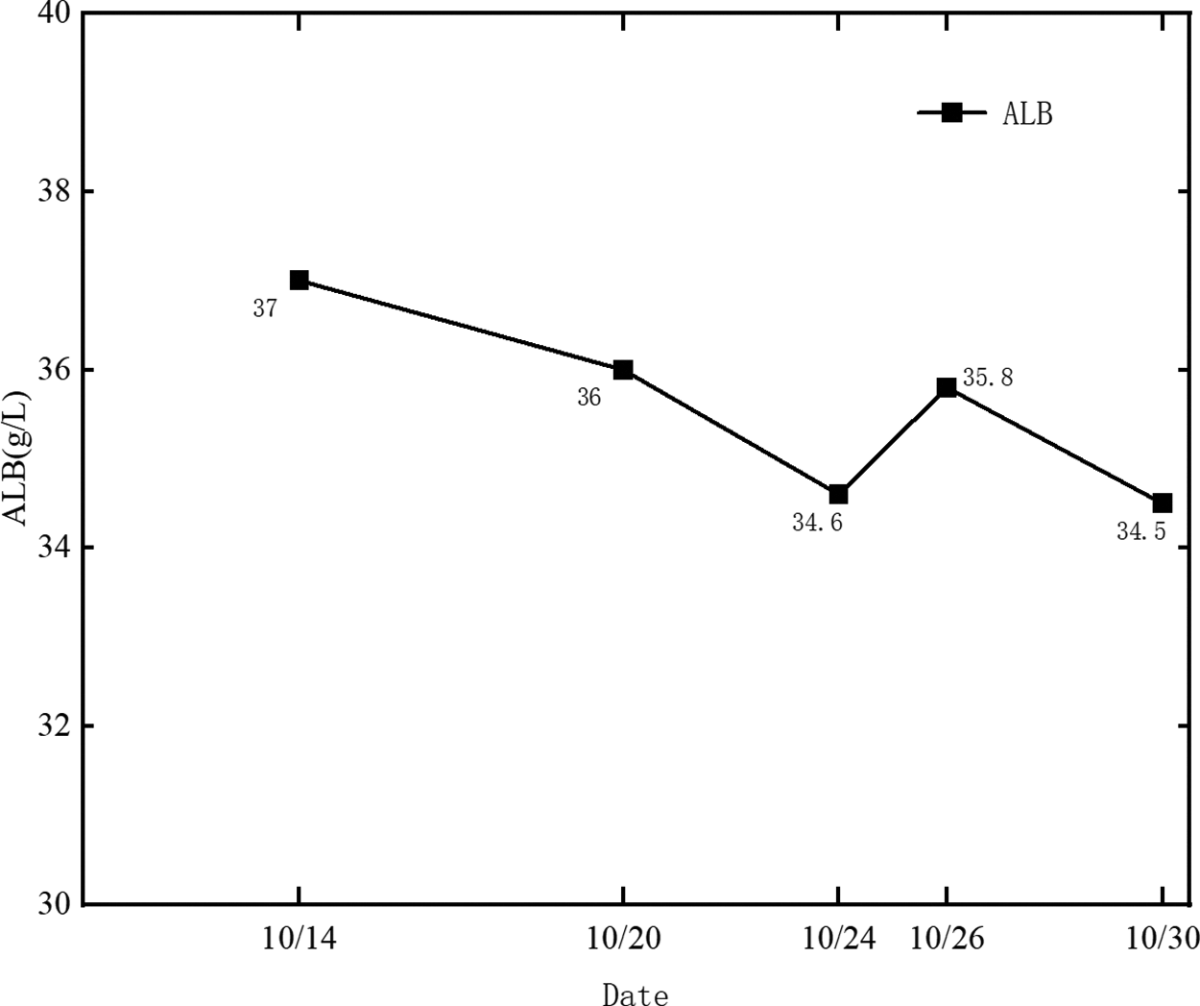
Albumin trends.

Routine blood tests showed a white blood cell count of 2.73–5.53 × 10^9^/L, an absolute neutrophil value of 2.03–3.87 × 10^9^/L, absolute lymphocyte value of 0.44–1.23 × 10^9^/L, and hemoglobin level of 86 to 119 g/L (Figs. 1 and 2).C-reactive protein was 5.63 to 108.53 mg/L (Figs. 1–3).Cardiac ultrasonography revealed mild mitral regurgitation. Primary screening for pathogens were all negative. Lung CT on October 12,2023 suggested scattered inflammation and interstitial changes in all lobes of the lungs, with ground-glass nodular shadows in the right upper-lobe of the lungs, and nodular shadows in the left lower lobe of the lungs (Fig. 4). Lung CT repeated on October 18,2023 suggested scattered inflammation and interstitial changes in both the lungs. In comparison to the previous film obtained on October 12,2023 an increase in the extent of localized inflammation in the upper-lobes of both lungs and the lower lobe of the left lung was noted (Fig. 5). Electronic fibreoptic bronchoscopy showed smooth mucosa and a patent lumen, and the anterior segment of the right upper-lobe bronchus was sampled for both transbronchial biopsy and bronchoalveolar lavage fluid (BALF). BALF was immediately stored at–80 °C and subjected to metagenomic NGS: nucleic acids were extracted from 300 µL using the Qiagen UCP Pathogen Mini Kit, libraries were prepared with Nextera XT, and sequencing was performed on an Illumina NovaSeq 6000 (2 × 150 bp, ~20 million reads/sample). Reads were trimmed with fastp, human sequences removed with Bowtie2 (hg38), and remaining reads classified by Kraken2 against the NCBI nt database. This analysis yielded 2717 unique Tropheryma whipplei reads (positive threshold ≥ 500 reads and ≥ 1 × coverage), with no other pathogens detected; accordingly, further histopathology was not pursued. Transbronchial biopsy of the same bronchial segment revealed no tuberculosis or neoplastic lesions, but did show focal alveolar epithelial hyperplasia and widened alveolar septa. The BALF cell differential was 90% macrophages, with a total nucleated cell count of 543 × 10^6/L. The liver function suggested a decrease in albumin levels (Fig. 6).

### 2.3. *Therapeutic intervention*

**Figure 7.**
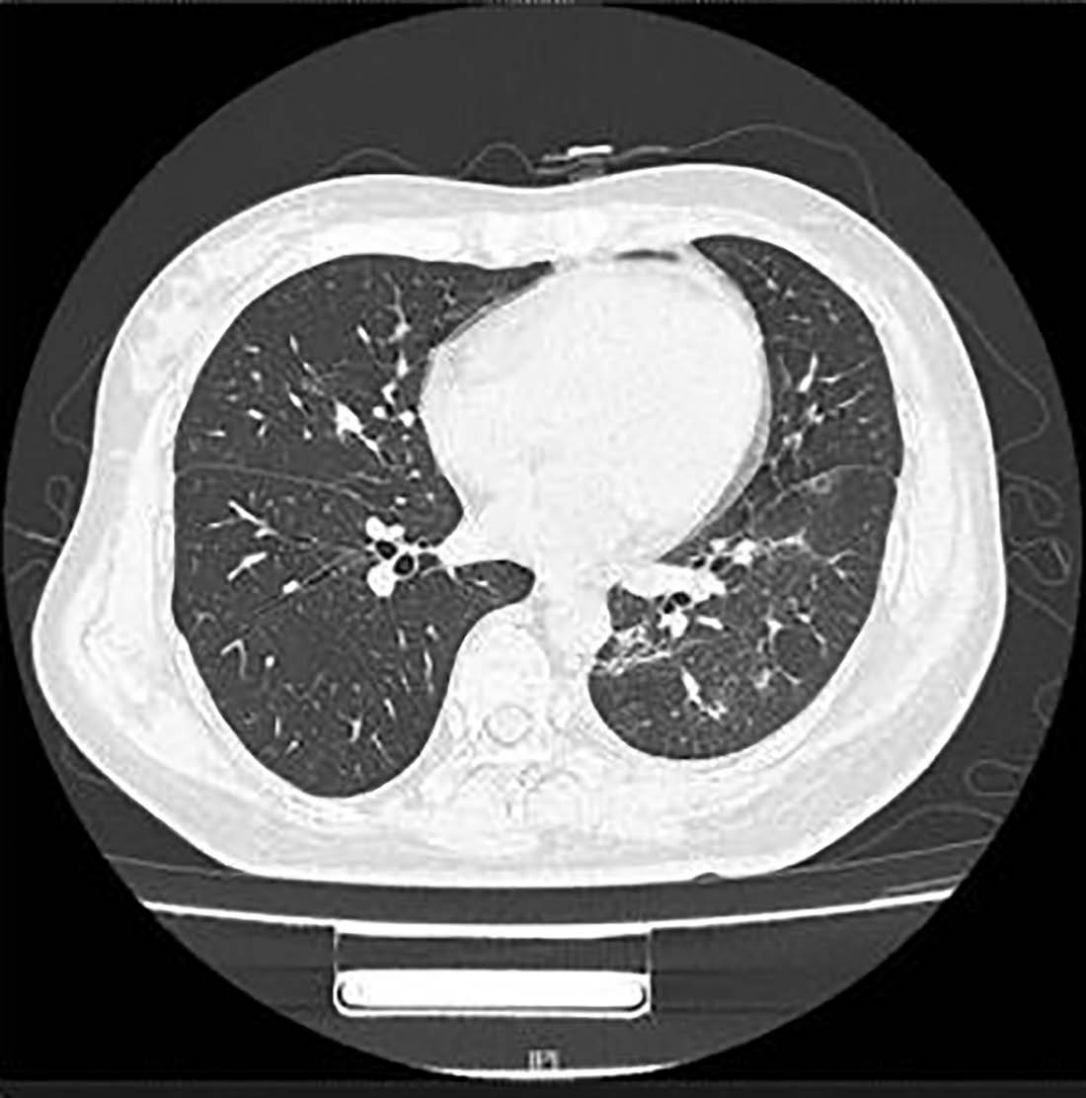
Patient was reexamined using pulmonary CT on October 30, 2023. CT = computed tomography.

Ampicillin and moxifloxacin were administered as empirical anti-infective treatments from October 13 to 20, 2023. However, the patient continued to experience fever, with a maximum temperature of 39.4°C. Symptomatic treatments such as cooling were administered; however, the temperature could not be lowered to normal levels. On 21st October, the patient was administered meropenem (1 g, 3 times per day), which reduced the patient’s fever frequency and maximum temperature. The maximum body temperature was lower than before; but in the blood routine review on 24th October, the total number of leukocytes was decreased to lower than the normal low value (Fig. 1) owing to an adverse drug reaction to meropenem. Subsequently, meropenem was replaced with doxycycline (0.1 g, day 2), and the patient’s body temperature gradually returned to normal, cough and phlegm symptoms alleviated, and leukocyte counts returned to normal. Lung CT was reviewed on 30^th^ October, suggesting scattered inflammation and interstitial changes in both lungs. The inflammation in the upper-lobe of the left lung was evidently absorbed, and the scope of localized inflammation in the upper lobe of the right lung and the lower lobe of the left lung were mildly enlarged; moreover, nodules were observed in the left lower lobe of the lung, which was considered as an inflammatory possibility (Fig. 7). The patient was discharged on November 1, 2023 with clinical improvement.

### 2.4. Follow-up and outcomes

**Figure 8.**
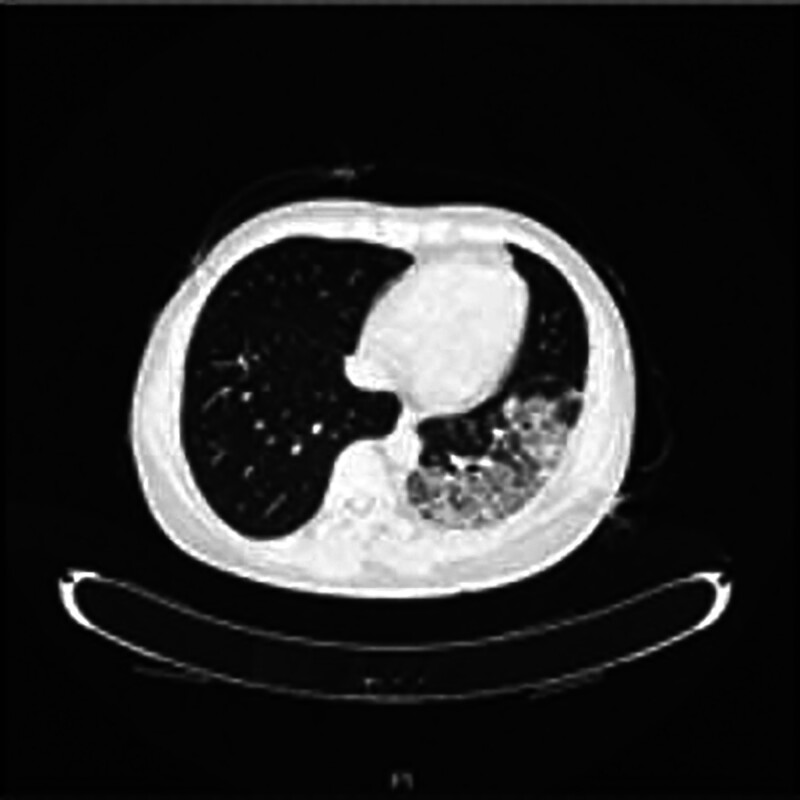
Patient was reexamined using pulmonary CT on November 14, 2023. CT = computed tomography.

**Figure 9.**
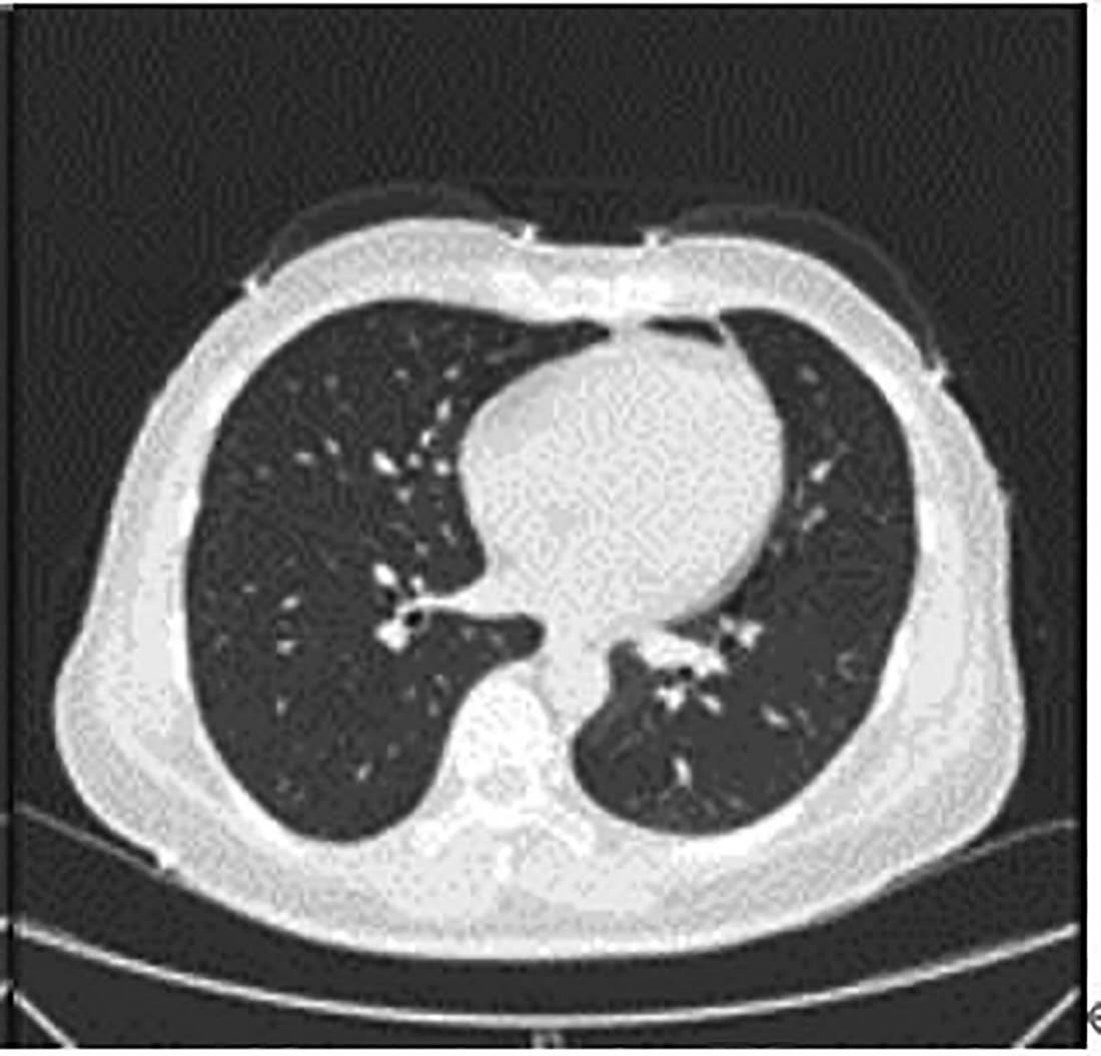
Patient was reexamined using pulmonary CT on January 11, 2024. CT = computed tomography.

**Figure 10.**
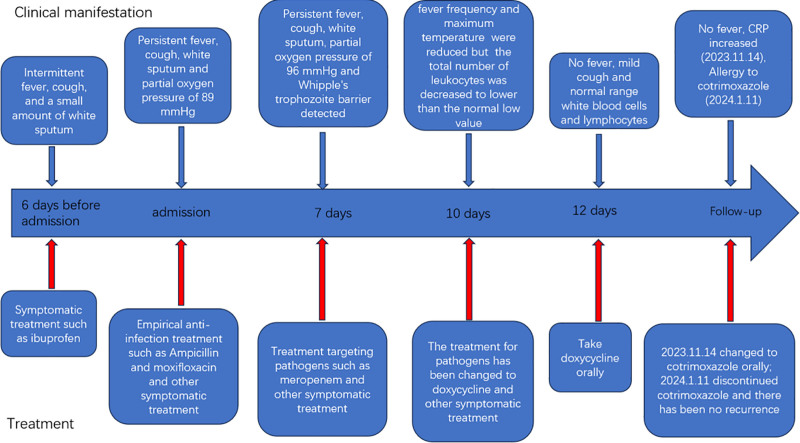
Clinical manifestations and treatment flowchart.

**Figure 11.**
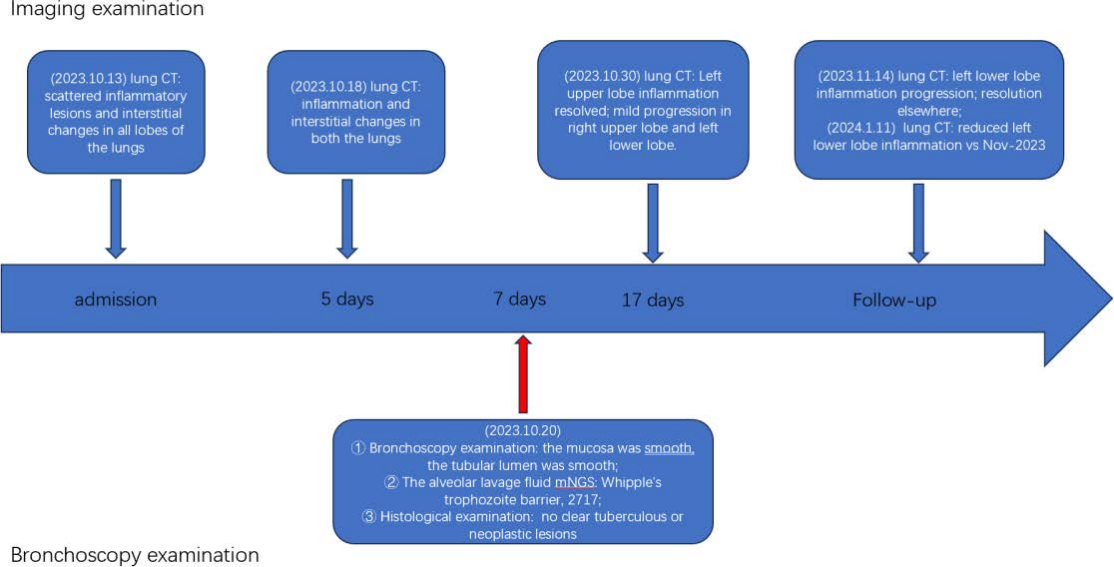
Flowchart of imaging and bronchoscopy results.

The patient was discharged from the hospital with regular oral doxycycline (0.1 g, day 2), during which there was no fever, occasional cough, or sputum symptoms. On November 14, 2023, the patient was reexamined in the outpatient clinic of our hospital, and the ultrasensitive C-reactive protein level was elevated (51.41 mg/L). Lung CT scans suggested that the extent of inflammation in the lower lobe of the left lung had increased, while the rest of the inflammation had decreased, as shown in Figure 8. Oral doxycycline was changed to co-trimoxazole and the patient was discharged from the hospital. On 14 December, the patient discontinued co-trimoxazole owing to a rash (allergic reaction), and lung CT was reviewed again on January 11, 2024 (Fig. 9), showing reduced inflammation in the upper and lower lobes of the left lung. Inflammation levels were reduced compared to the previous scan on November 14, 2023. All the clinical manifestations, diagnosis, treatment and examination results are summarized in Figures 10 and 11.

## 3. Discussion

Whipple trophoblast, recognized as an opportunistic pathogen, is predominantly transmitted via the fecal-oral route.^[4]^ This mode of transmission possibly accounts for the frequent involvement of the gastrointestinal (GI) tract in infections caused by this pathogen. Patients with GI tract involvement typically present with symptoms such as diarrhea, abdominal pain, and weight loss.^[2]^ In the present case, a detailed review of the patient’s medical history revealed no reported exposure to sewage or dirt. Additionally, the patient did not exhibit any symptoms indicative of digestive discomfort. Consequently, it is plausible that the route of infection in this patient was not digestive.

According to the available literature, the clinical symptoms of Whipple trophoblast infection vary^[5]^; however, acute lung infections are rare. The most common manifestations of Whipple trophoblast infection include anemia, elevated ultrasensitive C-reactive protein levels, and >50% of patients develop increased blood sedimentation and hypoproteinemia.^[6]^ These manifestations were present in our patient. Additionally, Whipple adoptive barrier is more likely to infect immunocompromised populations.^[7]^ In such patients, after unsuccessful screening for suspected pathogens and inadequate empirical anti-infective therapy, vigilance must be maintained for rare pathogenic infections. However, unlike patients with primary or secondary immunocompromise, the patient in this case had lymphoma, regular use of otuzumab, third cycle of maintenance treatment routine completed 2 months before the onset of the disease, and multiple sputum cultures that were negative. Imaging could not rule out lymphoma progression; moreover, the presence of targeted drug-associated pneumonia, coupled with the limitations of the traditional pathogenicity testing methods could not rule out the presence of primary lung infections. There has been remarkable advancements with the rapid development of genetic testing technology for the diagnosis of rare pathogenic infections.^[8]^ Therefore, when diagnosis is challenging, mNGS should be performed in a timely manner to obtain reliable pathogenetic evidence. In addition, Whipple trophoblast primarily infects macrophages. Whipple trophoblast enters the intestine and replicates in large numbers in intestinal mucosal macrophages.^[9]^ Furthermore, endoscopic mucosal biopsies are positive for macrophage glycogen staining, and Whipple trophoblast can be detected under electron microscopy. Therefore, the gold standard for Whipple trophoblast diagnosis is a pathological tissue biopsy.^[9]^ In this case, although a pathologic tissue biopsy was performed, no meaningful positive results were detected, and PAS staining was negative. However, the patient’s symptoms and related auxiliary test results improved significantly after administering treatment for this pathogen. Therefore, the main causative agent in this case was considered to be Whipple trophoblast. Notably, during the late follow-up period, the patient’s lung lesions exhibited migratory characteristics. Whipple disease in the joints causes migratory arthritis^[10]^; however, more cases are needed to verify whether the manifestation of Whipple disease in the lungs is migratory.

Effective anti-infective drugs include meropenem, ceftriaxone, co-trimoxazole, doxycycline, and hydroxychloroquine^[11]^; although the nucleic acid sequence of Whipple trophoblast was detected using macro-genetic sequencing, the patient’s pathologic biopsy did not reveal any positive results, and Whipple trophoblast rarely causes pneumonia. Hence, we could not confirm Whipple trophoblast as the causative agent. We used the relatively broad-spectrum meropenem for anti-infective treatment; however, owing to the adverse effects of meropenem, the patient was switched to doxycycline. Although meropenem causes leukocytes to decrease the incidence of this adverse reaction is low^[12]^; however, caution is advised, particularly in the immunocompromised population. After the administration of doxycycline, the patient’s symptoms improved, the white blood cell count returned to normal (Fig. 1), and the lung CT scan showed a resolution of the inflammation. Therefore, effective antibiotics against this pathogen will result in rapid symptomatic relief.^[13]^ The treatment of Whipple trophoblast remains controversial and there are no clear guidelines for its management.^[14]^ The prevalent treatment regimen used is based on retrospective analyses and prospective analyses,^[11]^which involves 2 weeks of induction therapy with intravenous ceftriaxone (2 g daily) followed by a switch to oral co-trimoxazole (960 mg twice daily) for 1 year. However, there are now reports on the ineffectiveness or relapse after the use of co-trimoxazole.^[9]^ Therefore, the use of co-trimoxazole is not recommended in the absence of neurological symptoms and a negative PCR for Whipple trophoblasts in cerebrospinal fluid. A new treatment regimen of doxycycline (100 mg twice a day) in combination with hydroxychloroquine (600 mg twice a day) for a total of 2 weeks can be considered.^[15]^ The patient’s clinical symptoms and lung CT improved with the use of doxycycline. Subsequently, the lesion appeared to wander and was accompanied by fluctuations in C-reactive protein. Hence, we used co-trimoxazole to alleviate the symptoms; however, owing to skin rash, we did administer the drug for long-term treatment. Moreover, as of the date of the submission of this report, the patient did not have any recurrence of the disease.

This study’s primary strength lies in documenting the first mNGS-diagnosed pulmonary Tropheryma whipplei infection in a lymphoma patient. Longitudinal imaging and laboratory data provide unique insights into therapeutic challenges in immunocompromised hosts, while unequivocally establishing mNGS as pivotal for diagnosing elusive pathogens. Limitations include its single-center case design, limiting generalizability, and truncated antibiotic therapy due to drug intolerance, precluding definitive long-term outcome assessment. Nevertheless, this case highlights critical diagnostic considerations for similar presentations.

## 4. Conclusion

The diagnostic and therapeutic experience of this case suggests that immunocompromised hosts are susceptible to infections with rare pathogens. When empirical anti-infective treatment reveals poor prognosis, mNGS examination of alveolar lavage fluid should be performed promptly to clarify the presence of these pathogens, and anti-infective treatment should be rapidly initiated accordingly. Whipple trophoblast may have a wandering manifestation in intrapulmonary lesions; however, this requires further verification in more cases. The treatment needs to be patient-specific. The course of treatment is relatively longer than that of common pneumonia, but it may not require a 1-year course of treatment; the treatment in this case lasted for 3 weeks, and the prognosis was good. Finally, more in-depth research on this bacterium is required to improve our understanding of this bacterium and to establish effective diagnosis and treatment guidelines.

## Acknowledgments

We extend our thanks to the Department of Respiratory and Critical Care Medicine at First Hospital of Jilin University for their valuable support. Thanks for the companionship and encouragement of Mr. Hou.

## Author contributions

**Data curation:** Langqing Xu.

**Formal analysis:** Langqing Xu.

**Investigation:** Langqing Xu.

**Software:** Langqing Xu.

**Validation:** Dan Li.

**Writing – original draft:** Langqing Xu.

**Writing – review & editing:** Dan Li.
